# The effects of using Tempeh as a supplement for type 2 diabetes

**DOI:** 10.1002/fsn3.3319

**Published:** 2023-03-17

**Authors:** Hui‐Kan Su, Wei‐Chao Chen, Jian‐He Lu, How‐Ran Chao, Yun‐Fang Liang, Sato Haruka, Wen‐Li Hsu, Mei‐Li Wu, Ming‐Hsien Tsai

**Affiliations:** ^1^ Department of Food Science, College of Agriculture National Pingtung University of Science and Technology Pingtung Taiwan; ^2^ Department of Pathology Laboratory Kaohsiung Veterans General Hospital Pingtung Branch Pingtung Taiwan; ^3^ Department of Environmental Science and Engineering, College of Engineering National Pingtung University of Science and Technology Pingtung Taiwan; ^4^ Emerging Compounds Research Center, General Research Service Center National Pingtung University of Science and Technology Pingtung Taiwan; ^5^ Department of Child Care, College of Humanities and Social Sciences National Pingtung University of Science and Technology Pingtung Taiwan; ^6^ School of Medicine, College of Medicine Kaohsiung Medical University Kaohsiung Taiwan; ^7^ Institute of Food Safety Management, College of Agriculture National Pingtung University of Science and Technology Pingtung Taiwan; ^8^ Department of Dermatology Kaohsiung Municipal Ta‐Tung Hospital, Kaohsiung Medical University Hospital, Kaohsiung Medical University Kaohsiung Taiwan; ^9^ Regenerative Medicine and Cell Therapy Research Center Kaohsiung Medical University Kaohsiung Taiwan; ^10^ Department of Oral Hygiene, College of Dental Medicine Kaohsiung Medical University Kaohsiung Taiwan

**Keywords:** type 2 diabetes, Tempeh

## Abstract

Studies suggest that the consumption of Tempeh can improve abnormal blood glucose and lipid parameters, although it remains still unclear as to whether Tempeh can improve tissue damage. In our study, db/db obese diabetic mice were given Tempeh 1 (300 mg/kg) and Tempeh 2 (600 mg/kg) for 3 months. The tissue samples collected were stained using different tissue‐staining methodologies and were compared with the diabetic control group that was not given any Tempeh. Our results demonstrated that consuming high‐dose Tempeh for 1 month could significantly reduce serum glucose and body weight in mice whereas the tissue section of our result could validate that consuming high‐dose Tempeh for 3 months effectively improves lipid droplet size and lipid accumulation in the liver, aorta, and kidney of the mice. Moreover, an indication of the recovery of the damaged tissue could be observed in the heart and pancreatic tissue when high dosage of Tempeh was given as a treatment. Thus, it can be concluded that continuous consumption of Tempeh as a treatment could improve both blood glucose and body weight of diabetic mice while also improving lipid accumulation and tissue damage.

## Graphical abstract

Continuous consumption of Tempeh as a treatment can improve both the blood glucose and body weight of diabetic rats while also improving lipid accumulation and tissue damage.

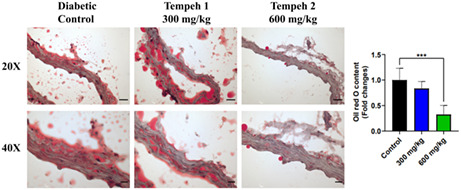

## 1 INTRODUCTION

Societal advancements have allowed economic progression, changes in the general public's lifestyle, and dietary patterns. Easily accessible high‐caloric, fat, and sugar foods have increased the prevalence of obesity and metabolic syndromes among the population today with the consequent increase in the risk of being diagnosed with diabetes, specifically type 2 diabetes, which is a global epidemic today (Schmidt, 2018). In 2014 alone, 9.3% of Americans were diagnosed with diabetes, and 40% were at a lifetime risk of developing diabetes (Schmidt, 2018). Statistics state that there were 1.8 billion diabetic patients globally in 2014, which is double the number in 2000 (American Diabetes, 2014). Scholars estimate that not only will diabetic patients around the world increase to 5.92 billion by 2035, but specifically in Asian countries, 42.3 million people will be diagnosed with diabetes, which is a 21.5 million increase compared to the year 2000 (Guariguata et al., 2014; Yang et al., 2012).

Nevertheless, studies have proven that a controlled diet is able to impede the development of type 2 diabetes (Sami et al., 2017); specifically, fermented soybean products are suggested to slow or prevent its progression (Kwon et al., 2010). Tempeh is a fermented traditional soybean product that originated in Indonesia. During the fermentation process, the nutrients and vitamins in soybeans, such as free amino acids, fatty acids, oligosaccharides, nicotinic acid, flavonoids, pantothenic acid, and vitamins B1, B2, B6, B12, and vitamin D, will go through changes for the benefit of one's health. Tempeh is also abundant in isoflavones compared to other soy products, which allow the production of phytochemicals through organismal metabolism (Bisping et al., 1993; Keuth & Bisping, 1993; Reu et al., 1994; Watanabe et al., 2006). Moreover, the free fatty acids in Tempeh inhibit the activity of hydroxymethylglutaryl CoA (HMG‐CoA) reductase, which is a key synthetic enzyme for cholesterol in the liver (Hermosilla et al., 1993).

In order to investigate the result of Tempeh on diabetes, hyperlipidemically induced mice were treated with Tempeh for 2 months (Astuti et al., 2000). The experimental results proved that Tempeh was able to reduce the total cholesterol and triglycerides in the serum, thus significantly reducing the cholesterol content in the liver, thereby improving the lipid oxidation state in both the serum and the liver (Astuti et al., 2000). Similarly, in another study, a significant increase in high‐density lipoprotein while reducing the total cholesterol, triglycerides, and low‐density lipoprotein (LDL) in the serum of mice that consumed Tempeh over 6 weeks was noticed (Watanabe et al., 2006); moreover, when Tempeh was given to adult patients with hypercholesterolemia for 3 months, the triglycerides in the liver significantly decreased. In brief, LDL values and lipid oxidation levels (MDA concentration) were all reduced regardless of gender (Astuti et al., 2000). Accordingly, Huang et al. (2018) evaluated the effects of Tempeh cofermented with lactic acid bacteria on glucose. Additionally, cholesterol synthesis was inhibited while intestinal microflora and fat metabolism regulation were promoted (Huang et al., 2018).

The current research focuses on the improvement of Tempeh for blood biochemical levels. As it is still unclear whether Tempeh is able to improve tissue damage, in this investigation, db/db obese diabetic mouse and tissue‐staining methodologies were used to explore visceral tissue improvements when Tempeh was given to assist blood glucose control in type 2 diabetes.

## 2 TEMPEH PREPARATION: MATERIALS AND METHODS

Kaohsiung No. 9 soybeans were fermented to produce Tempeh products. Before fermentation, the soybeans were washed, peeled, and soaked in water for 12 h, then after 12 h, they were dried and immersed in deionized water that was twice the original weight of the soybeans. The pH of the deionized water was then adjusted to pH 3.5–4.0 with 1% lactic acid, and the mixture was boiled at 100°C for 30 min. *Rhizopus oligosporus (R. oligosporus)* spores were added to the sample after it being cooled to room temperature for fermentation. Once added, the sample was placed in the incubator at a temperature between 32 and 35°C for 48 h to ferment until the hyphae covered the soybeans. The fermented product was then placed in an oven at 65°C and dried with hot air, then the tempeh samples were sterilized with Ultraviolet irradiation for 12 h and finally ground into powder and stored in a refrigerator at −20°C for later use.

### 2.1 Animal preparation: Environment and diets

Eighteen 8‐week‐old male BKS.Cg‐Dock7^m^ +/+ Lepr^db^/JNarl (db/db) mice were obtained from BioLASCO Taiwan Co., Ltd. The mice were maintained at a temperature of 25 ± 2°C with a relative humidity of 55%–60% alternating with a light/dark cycle for 12 h at the National Pingtung University of Science and Technology. All mice were fed the Laboratory Autoclavable Rodent Diet 5010 diet (LabDiet 5010) ad libitum with free access to drinking water at all times. After a week of adaptation, the mice were randomly assigned to three groups of six and were fed with different concentrations of Tempeh or without Tempeh by oral gavage for 12 weeks. Group 1 was administered 300 mg/kg Tempeh, group 2 was administered 600 mg/kg Tempeh, and the diabetic control group was administered 600 mg/kg casein. Body weight and blood glucose level, taken by tail puncture and measured using test strips, were measured weekly during the experimental period (Accu‐Chek Active; Roche Diagnostics), then the mice were anesthetized at the end of the trial. This protocol was approved by the Ethics Committee at the National Pingtung University of Science and Technology.

### 2.2 Histopathological analysis on the tissues samples

Mice tissue specimens were processed according to the standard hematoxylin and eosin (H&E) staining technique. To begin with the analysis, harvested tissue samples were fixed in a 4% formaldehyde, dehydrated with ethanol, and embedded in paraffin, then cut and mounted on a glass slide. Before staining, tissue samples were deparaffinized and rehydrated with 100% xylene. Once rehydrated, the sections were dehydrated in graded concentrations of ethanol (100%, 95%, 75%, and 50%) and washed with distilled water before it was stained. The nuclei were first stained with hematoxylin (Sigma‐Aldrich), then washed with distilled water, and dipped in 0.3% acid alcohol (Sigma‐Aldrich). Next, the samples were washed with distilled water before the sections were stained with eosin (Sigma‐Aldrich) again followed by dehydration with ethanol and finally fixed with a cover glass. After the staining, the tissue was observed under an optical microscope.

### 2.3 Oil red O staining

The tissue slices were first treated with sodium oleate for 24 h, then washed with phosphate buffer saline twice before 4% paraformaldehyde was added to the slices. Then, the slices were washed with H_2_O and treated with 0.05%‐TritonX‐100 for 10 min before being soaked in 60% isopropyl alcohol. Finally, the tissue slices were stained using 60% Oil red O solution for 30 min and washed with 60% isopropyl alcohol, and observed under the microscope.

### 2.4 Statistical analysis

All experimental results were recorded with means ± SD. The differences between the diabetic control and Tempeh‐treated groups were analyzed by one‐way analysis of variance (ANOVA) and Scheffe's multiple range tests (IBM SPSS Statistics 21) with a significance threshold of *p* < .05.

## 3 RESULTS AND DISCUSSION

Patients diagnosed with type 2 diabetes exhibit weight gain associated with persistent hyperglycemia (Boudina & Abel, 2010; Poornima et al., 2006). As a model of type 2 diabetes, db/db mice exhibit similar characteristics such as spontaneous weight gain and elevated fasting blood glucose concentration (Belke et al., 2000). Studies have shown that the db/db mice will demonstrate signs of weight gain and persistent hyperglycemia at 6 weeks of age (Dludla et al., 2017).

### 3.1 Effects of tempeh treatments on weight and blood glucose in db/db mice

**FIGURE 1:**
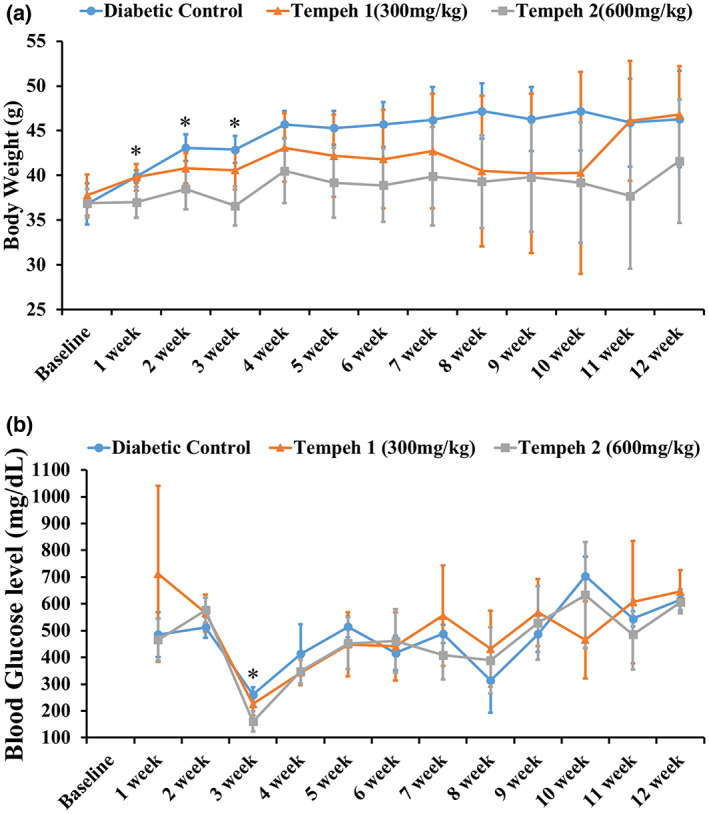
Tempeh 2 (600 mg/kg) reduced the most body weight and blood glucose level of the db/db mice compared to the diabetic control and Tempeh 1 (300 mg/kg) at week 3. (a) The weight of the mice at weeks 1, 2, and 3 was significantly lower than the diabetic controls, demonstrating that the Tempeh 2 treatment was the most effective during the first 3 weeks. (b) The blood glucose level of the mice at week 3 was significantly lower than the diabetic control, demonstrating that the Tempeh 2 treatment was the most effective. Diabetic Control: db/db mice with normal diet; Tempeh 1: db/db mice with normal diet + Tempeh (300 mg/kg body weight); Tempeh 2: db/db mice with normal diet + Tempeh (600 mg/kg body weight). Results: mean ± SD, *p < .05 (assessed by ANOVA).

The body weight and blood glucose of the mice were measured every week during the experiment (Figure 1), with the weekly weight revealing that the diabetic control group had a higher average body weight compared to the group that was fed Tempeh. In detail, after a week of feeding, the average body weight of the group 2 Tempeh treatment mice (37.0 ± 1.7) that was fed with more Tempeh had a significant reduction in weight with *p* = .010 in comparison with the diabetic control group (39.9 ± 0.7) and group 1 Tempeh treatment mice (39.8 ± 1.5). More succinctly, as the weight of the mice in the diabetic control group continued to increase, group 2 Tempeh treatment group maintained significantly lower body weight. In the data from the second week, although the group 1 Tempeh treatment group's mice also showed a lower average weight compared to the diabetic control group, the result was not statistically significant. As a result, this experiment demonstrated that the feeding protocol of 600 mg/kg of Tempeh could effectively prevent weight gain of the db/db mice.

While the db/db mice showed persistent hyperglycemia at the beginning of the experiment, the results revealed that after feeding the mice Tempeh for 3 weeks continuously, group 2 Tempeh treatment (160.8 ± 37.6) had significantly lower blood glucose (*p* = .003) compared to the diabetic control group (260.8 ± 27.8). In brief, the result indicates that feeding Tempeh 600 mg/kg could effectively reduce blood glucose levels.

### 3.2 The effects of tempeh on lipid droplet size and lipid accumulation reduction in the liver

Obesity and insulin resistance in type 2 diabetes result in abnormal lipid metabolism, where fatty acids might accumulate in the liver, adipose tissue, and skeletal muscle, as well as the development of insulin resistance (Cornier et al., 2008). Insulin resistance in the skeletal muscle will promote the liver to convert excessive blood glucose into fatty acids through the effect of de novo lipogenesis (Flannery et al., 2012). Excessive fatty acids are synthesized into triglycerides and stored in the liver cells and hepatocytes, in the form of lipid droplets, which will further develop into steatohepatitis (Gluchowski et al., 2017).

**FIGURE 2:**
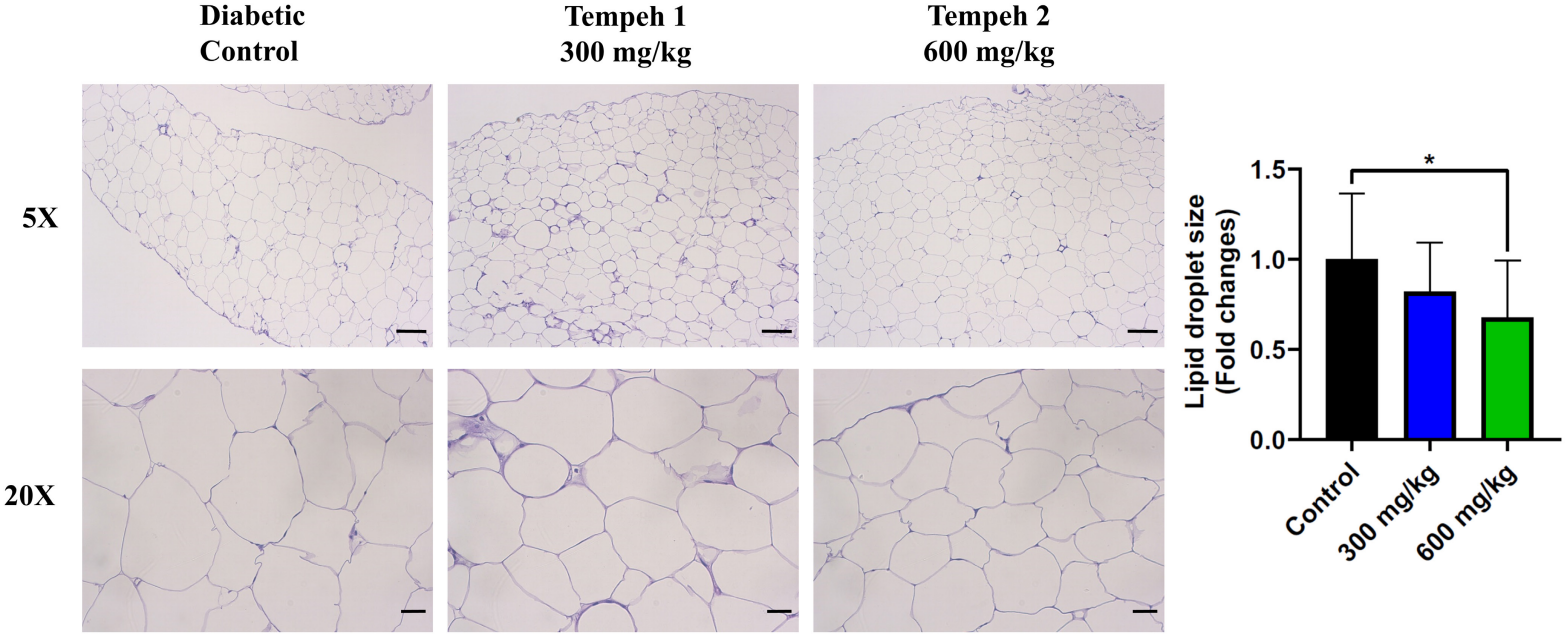
Tempeh 2 (600 mg/kg) reduced the size of the lipid droplet in the db/db mice the most when in comparison with the diabetic control and Tempeh 1 (300 mg/kg). The lipid droplets in the adipocytes of the db/db mice were observed with H&E staining. While the difference in lipid droplet size appears less noticeable under 5× microscope, the reduced lipid droplet size was evident under 20× microscopy. Magnifications: 5× (upper panel; scale bar = 100 μm) and 20× (lower panel; scale bar = 20 μm). Right panel: comparison of the lipid droplet size between each group (mean ± SD, *p < .05).

**FIGURE 3:**
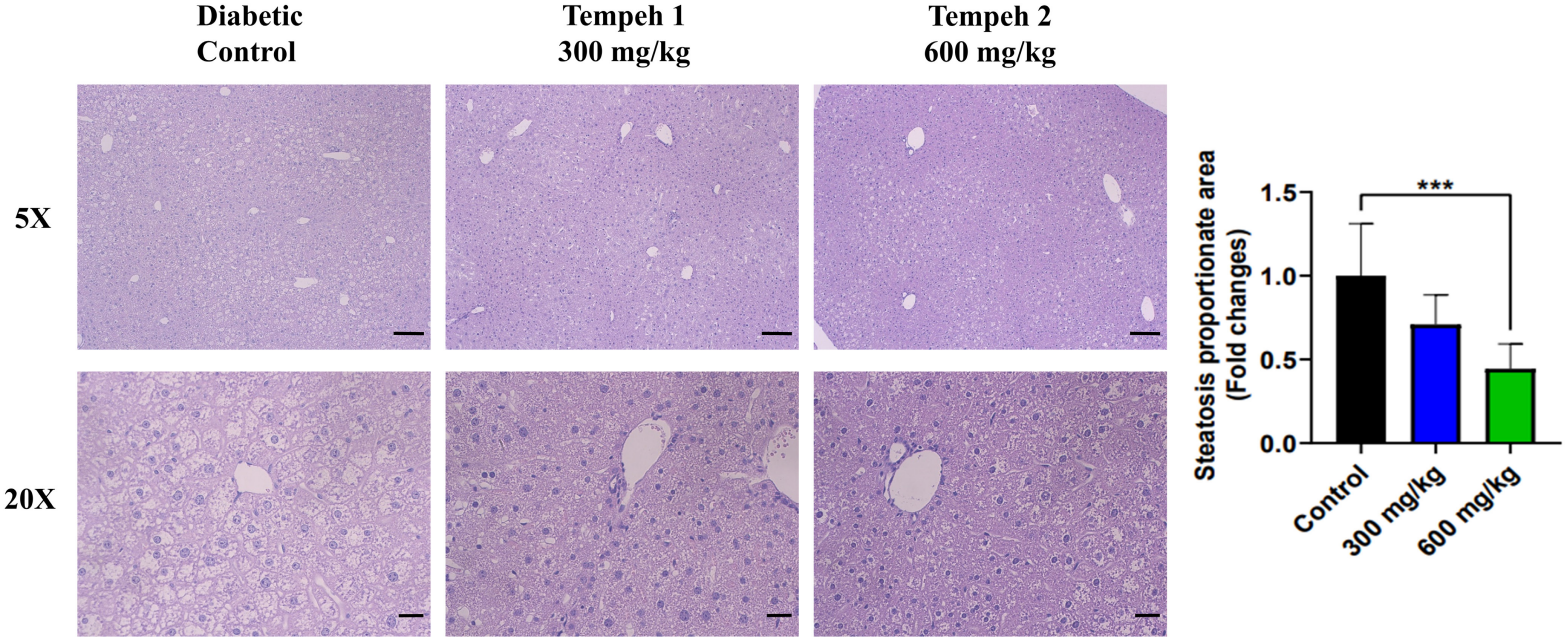
Tempeh 2 (600 mg/kg) reduced the size of the hepatic steatosis in the db/db mice the most when in comparison with the diabetic control and Tempeh 1 (300 mg/kg). Hepatic steatosis in the liver was investigated with H&E staining. At 20× magnification, less white areas around the cells can be observed, demonstrating that Tempeh 2 treatment reduced the most lipid. Magnifications: 5× (upper panel; scale bar = 100 μm) and 20× (lower panel; scale bar = 20 μm). Right panel: comparison of the steatosis proportionate area between each group (mean ± SD, ***p < .001).

In order to detect the changes in adipose tissue and liver tissue of db/db mice after feeding Tempeh for 12 weeks, H&E staining was used to observe the samples (Figures 2 and 3). It was found that while adipose tissue of the diabetic control group showed a larger lipid droplet size, after 12 weeks of Tempeh, the droplet size was significantly reduced. The reduced droplet size in the group 2 600 mg/kg Tempeh treatment was more evident than that from the group 1 300 mg/kg Tempeh treatment (Figure 2). Further quantification of lipid droplet size also found that the average area of lipid droplets decreased after 12 weeks of Tempeh administration, and the average area of lipid droplets in Group 2 (600 mg/kg Tempeh; 1619.2 ± 520.5 μm^2^) was significantly lower than that in the diabetic control group (2157.9 ± 81.1 μm^2^) (Figure S1).

Moreover, morphological changes in the liver of the db/db mice due to lipid droplet accumulation caused pale discoloration on the tissue slides; the ratio of pale discoloration (steatosis proportionate area) was significantly decreased with Tempeh treatment (Figure 3). In summary, the lipid droplet size in adipose tissue and lipid accumulation of the liver in db/db mice were significantly reduced after feeding Tempeh as a treatment for 3 months.

### 3.3 The effects of tempeh on fat accumulation in the aorta and pathomorphism of the heart tissues in the db/db mice

Abnormalities in cardiovascular and heart tissues are common complications in diabetic patients. For instance, steatohepatitis and atherosclerosis, as a result of hyperglycemia, are some risk factors for various cardiovascular complications (Byrne & Targher, 2015; Ren et al., 2016). It is also known that diabetic cardiomyopathy (DCM) is one of the leading complications and causes of cardiovascular disease, which is led by other factors aforementioned (Hamby et al., 1974; Yang et al., 2016). DCM might cause myocardium deterioration and enlargement of the left ventricular mass while also reducing diastolic function (Fujiwara et al., 2015; Miki et al., 2013). In 2014, studies estimated that the prevalence rate of DCM in diabetes patients was 16.9% and type 2 DCM was also one of the main causes of death in such patients (Chang et al., 2015; Dandamudi et al., 2014).

**FIGURE 4:**
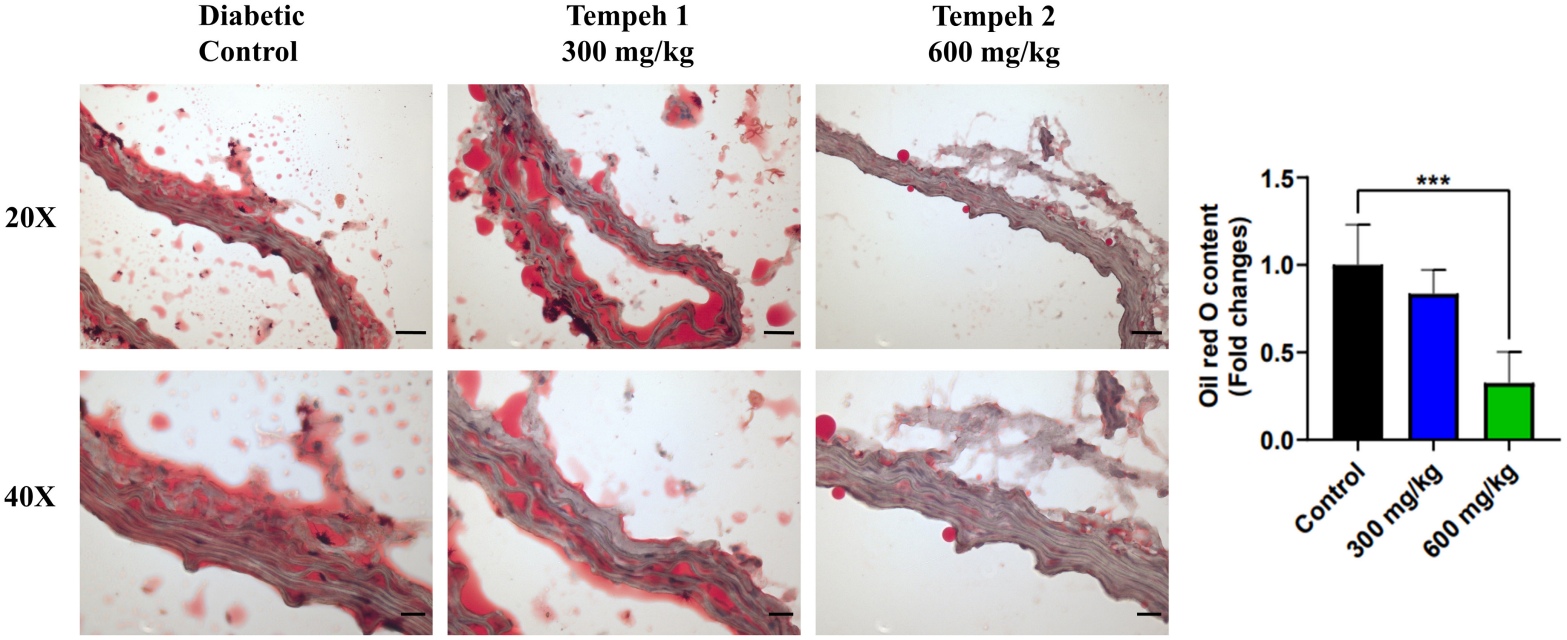
Tempeh 2 (600 mg/kg) was the most effective in ameliorating the fat accumulation in the aorta of the db/db mice when in comparison with the diabetic control and Tempeh 1 (300 mg/kg). Oil red O staining was used to observe the fat in the aorta of the db/db mice. It is evident that after treatment with 600 mg/kg Tempeh, less red area can be observed on the slices, demonstrating that fat in the aorta was reduced. Magnifications: 20× (upper panel; scale bar = 20 μm) and 40× (lower panel; scale bar = 10 μm). Right panel: comparison of the Oil red O content between each group (mean ± SD, ***p < .001).

**FIGURE 5:**
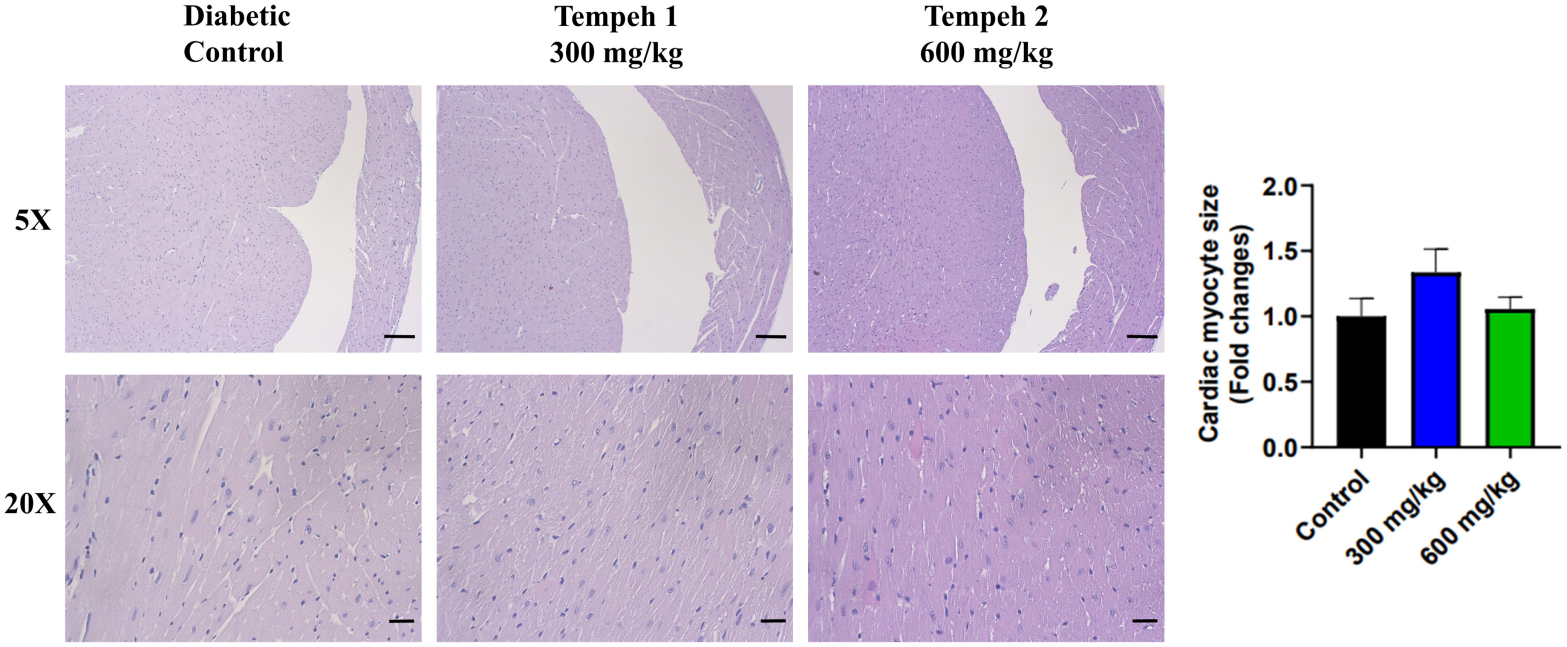
The heart tissue sample from Tempeh 2 (600 mg/kg) was more compact compared to the diabetic control and Tempeh 1 (300 mg/kg). H&E staining was used to observe the pathological changes of the db/db mice heart tissues. The heart tissues of both the diabetic control group and Tempeh 1 group were damaged due to prolonged hyperglycemia. However, an indication of heart tissue recovery from the Tempeh 2 group can be observed as a result of higher Tempeh treatment, resulting in more compacted tissue. Magnifications: 5× (upper panel; scale bar = 100 μm) and 20× (lower panel; scale bar = 20 μm). Right panel: comparison of the cardiac myocyte size between each group (mean ± SD).

With the Oil red O staining to observe the fat accumulation, it was found that treatment with Tempeh in the db/db mice reduced their aortic fat accumulation in a dose‐dependent manner, especially after feeding 600 mg/kg for 3 months showing statistical significance (Figure 4). Besides, it was observed in the H&E staining that the diabetically controlled mice group had cardiac hypertrophy and fibrous disorders (Figure 5). After 3 months of Tempeh administration, the condition had slightly improved, yet the improvement was only slightly overt.

### 3.4 Effects of tempeh on the pathomorphism of the kidney in the db/db mice

Being in a state of hyperglycemia for a period of time can cause tissue damage and multiple complications, for instance, diabetic kidney disease. In the early stages, high blood glucose will increase the glomerular filtration rate, which leads to glomerular hypertrophy, proliferation, and pathological changes (Alicic et al., 2017; Premaratne et al., 2015). In later stages, the damaged kidney results in dysfunctional kidney filtering, so harmful substances cannot be effectively removed, which will lead to the increase of blood urea nitrogen and creatinine, and eventually, to kidney failure (Alicic et al., 2017). In other words, diabetic kidney disease is a leading cause of end‐stage renal disease and an important factor in causing dialysis.

**FIGURE 6:**
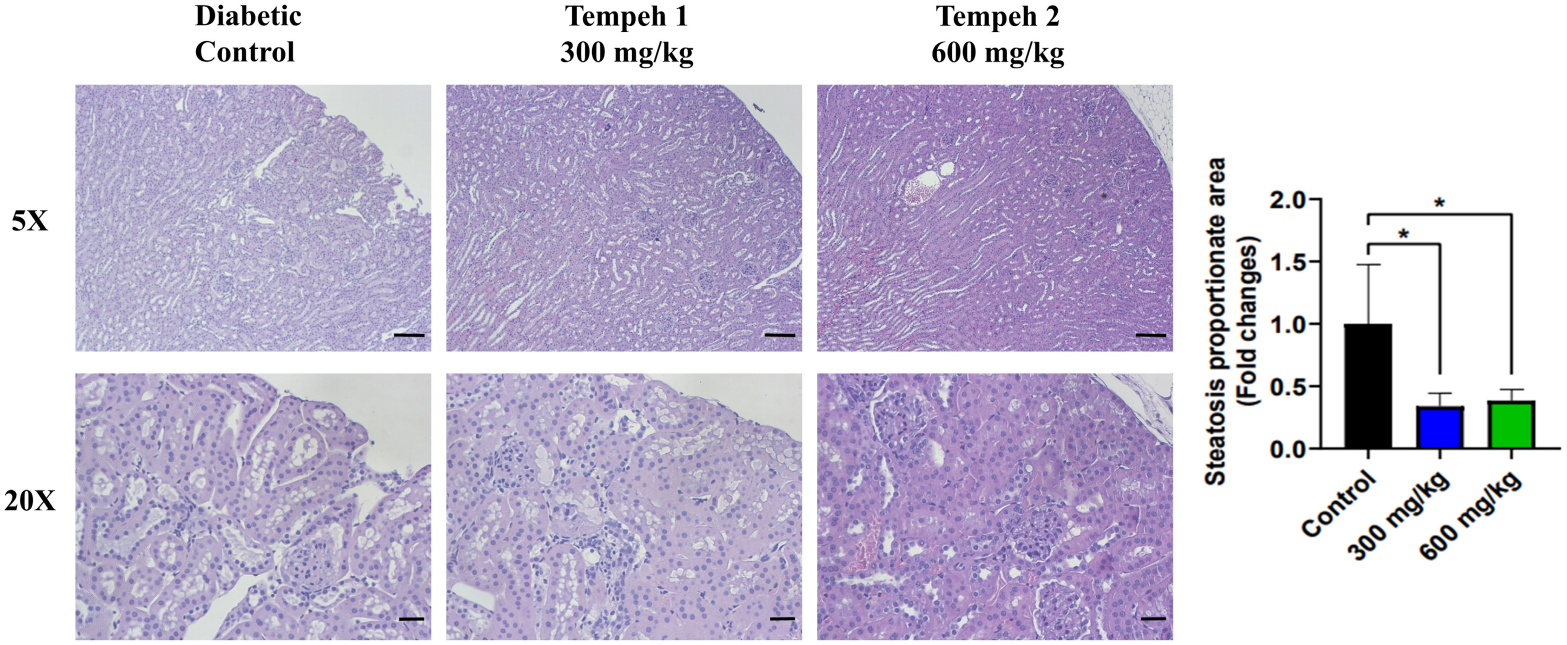
The kidney tissue sample from Tempeh 2 (600 mg/kg) was more compact compared to the diabetic control and Tempeh 1 (300 mg/kg). H&E staining was used to observe the pathological changes of the db/db mice kidney tissues. Tempeh 2 group with a higher dose of Tempeh allowed the recovery of damaged tissue due to prolonged hyperglycemia as the tissue was more closely packed. Magnifications: 5× (upper panel; scale bar = 100 μm) and 20× (lower panel; scale bar = 20 μm). Right panel: comparison of the steatosis proportionate area between each group (mean ± SD, *p < .05).

In this experiment, the effect of Tempeh on damaged kidney tissue caused by hyperglycemia was evaluated. The db/db mice kidneys in the diabetic control group were continuously exposed to hyperglycemia throughout the experiment. The experimental result from the H&E staining revealed severe lipid deposition in renal tubules, and 3 months after Tempeh administration, the lipid deposition was significantly alleviated (Figure 6). We also observed that the value of serum creatinine was decreased by Tempeh treatment, but there was no statistically significant difference between the diabetic control and Tempeh‐fed groups (Figure S2). Taken together, supplemented treatment with Tempeh in the db/db mice efficiently attenuated the lipid accumulation in tissues.

### 3.5 The effects of tempeh on the db/db mice pancreatic pathomorphism and spleen tissues in the db/db mice

**FIGURE 7:**
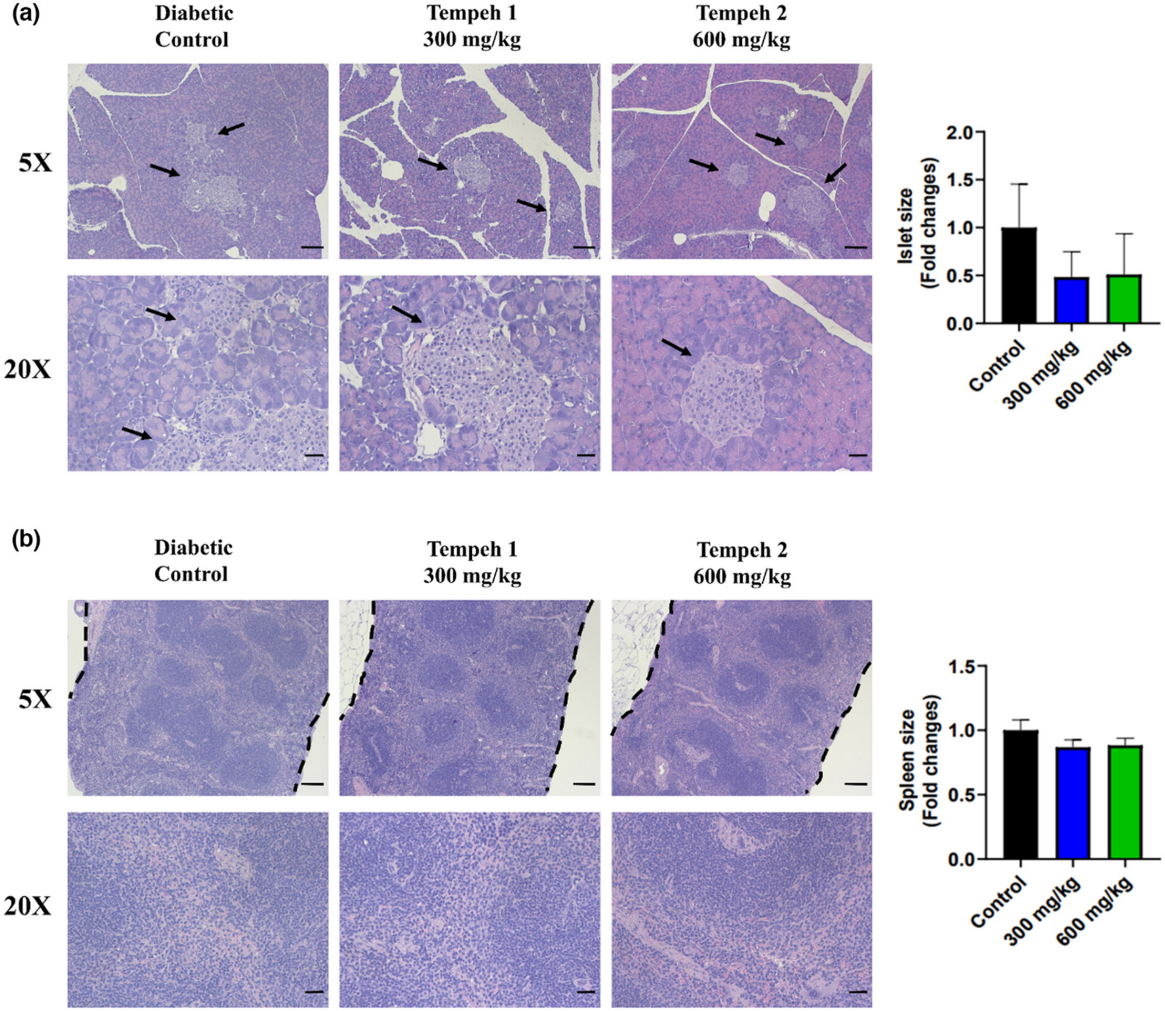
The pancreas and spleen tissue samples from Tempeh 2 (600 mg/kg) were more compact compared to the diabetic control and Tempeh 1 (300 mg/kg). H&E staining was used to observe the pathological changes of the db/db mice pancreas and spleen tissues. Higher dose of Tempeh (600 mg/kg) recovered the damaged tissues of the db/db mice. Magnifications: 5× (upper panel; scale bar = 100 μm) and 20× (lower panel; scale bar = 20 μm). Right panel: comparison of the islet and spleen sizes between each group (mean ± SD). Black arrows indicate islets.

The effects of Tempeh on the alleviation of db/db mice pancreas and spleen tissue damage were also evaluated in this experiment. H&E staining showed that the islets of the diabetic control group were abnormally large and the tissues were loosely distributed (Figure 7). When in comparison with the diabetic control group, the islets of 600 mg/kg mice showed a decreasing trend, and the tissues were also tighter. Additionally, the spleen was enlarged in the diabetic control group at low magnification, but no difference was observed in the groups that used Tempeh as treatment.

In the United States, the nutritional content per 84 g of commercial Tempeh includes 14.5 ± 2.4 g protein, 7.0 ± 4.6 g fiber, 0.6 ± 0.7 g saturated fat, 0.8 ± 1.3 g sugar, 14.2 ± 6.3 g carbohydrate, 152.5 ± 15.9 cal energy, 64.4 ± 22.3 mg calcium, 153.8 ± 151.2 mg potassium, 0.8 ± 1.1 g monounsaturated fatty acids, and 1.2 ± 1.6 g polyunsaturated fatty acids, all being free of cholesterol as well as trans fatty acids (Ahnan‐Winarno et al., 2021). Our previous clinical study results suggested that 2‐g Tempeh daily for a period of 3 months attenuated HbA1c and triglyceride levels in participants (Su et al., 2021). One animal study suggested that a high‐grade protein and fiber diet could remodel the gut microbiota, resulting in improved glycemic control and ameliorated diabetes (Ni et al., 2022). In addition, isoflavone content, polyphenol content, and antioxidant activity were increased during the fermentation of Tempeh (Kuligowski et al., 2017). The current study showed that total polyphenolic content (6157 mg GAE/kg vs. 1203 mg GAE/kg in the soybean), gallic acid (21.70 ± 1.85 mg/kg vs. 1.58 ± 0.44 mg/kg in the soybean), daidzein (420 mg/kg vs. 49 mg/kg in the soybean), and genistein (310 mg/kg vs. 63 mg/kg in the soybean) were increased in the Tempeh group compared with the soybean group. However, the content of catechin (75.11 ± 2.24 mg/kg vs. 96.34 ± 6.14 mg/kg in the soybean), caffeic acid (16.46 ± 0.45 mg/kg vs. 20.93 ± 2.23 mg/kg in the soybean), rutin (3.88 ± 0.21 mg/kg vs. 11.59 ± 0.89 mg/kg in the soybean), naringin (101.42 ± 5.19 mg/kg vs. 128.86 ± 11.63 mg/kg in the soybean), daidzin (667 mg/kg vs. 1050 mg/kg in the soybean), and genistin (788 mg/kg vs. 948 mg/kg in the soybean) was reduced in the Tempeh group compared with the soybean group (Table S1). Furthermore, the antioxidant capacity was also increased in the Tempeh group compared with the soybean group (Table S2).

In many animal and cellular models, isoflavones have effectively demonstrated their ability to activate human islet β‐cell proliferation, protect islet β‐cells, and improve insulin secretion (Duru et al., 2018). In type 2 diabetes mice, polyphenol extracts reduced serum alanine aminotransferase (ALT) and aspartate transaminase (AST) activities, inhibited inflammatory responses, and regulated intestinal microflora, which improved the expression of type 2 diabetes (Shen et al., 2022). Together, the improvement of type 2 diabetes may be related to the nutritional composition and biological activity of Tempeh.

## 4 CONCLUSION

In this study, variant doses of Tempeh treatment were given to db/db mice for 3 months to observe the tissue damage caused by diabetes. The results demonstrated that after 3 weeks of Tempeh 600 mg/kg administration, mice weight gain and blood glucose rise were both inhibited; furthermore, after 3 months of continuous administration, lipid droplet size was reduced in the aorta, and lipid accumulation was inhibited in the liver and kidney. Although there were slight improvements in the heart and pancreatic tissue, the improvements were apparent; hence, we recommend that Tempeh should be taken continuously as a supplement to improve the damage induced by type 2 diabetes.

## CONFLICT OF INTEREST STATEMENT

The authors declare that they do not have any conflict of interest.

## ETHICS STATEMENT

The animal study protocol was approved by the Ethics Committee at the National Pingtung University of Science and Technology (NPUST‐109‐079).

## Supporting information


Appendix S1

## Data Availability

The data that supports the findings of this study are available within the article and its supplementary materials.
