# Supplementary material for: The effects of using Tempeh as a supplement for type 2 diabetes
Source: Food Sci Nutr. 2023 Mar 17;11(6):3339–47. doi: 10.1002/fsn3.3319 (PMC10261801; doi:10.1002/fsn3.3319)
Supplement: Supplementary file 1 — Appendix S1 [file FSN3-11-3339-s001.docx]

**SUPPORTING INFORMATION**

**The effects of using Tempeh as supplement for Type II diabetes**


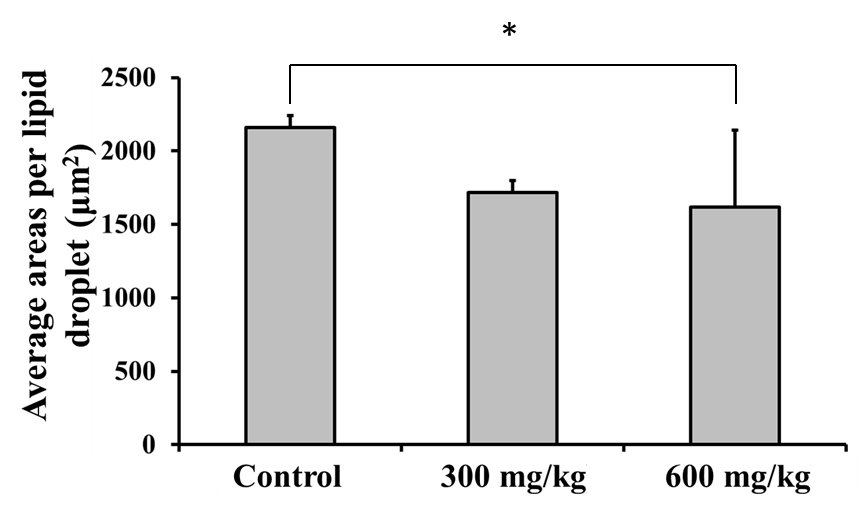


**SUPPORTING INFORMATION FIGURE S1** Treatment with Tempeh 600mg/kg reduced the average areas per lipid droplet in adipocytes of the db/db mice in comparison with the diabetically controlled group (mean ± SD, **P* < 0.05).


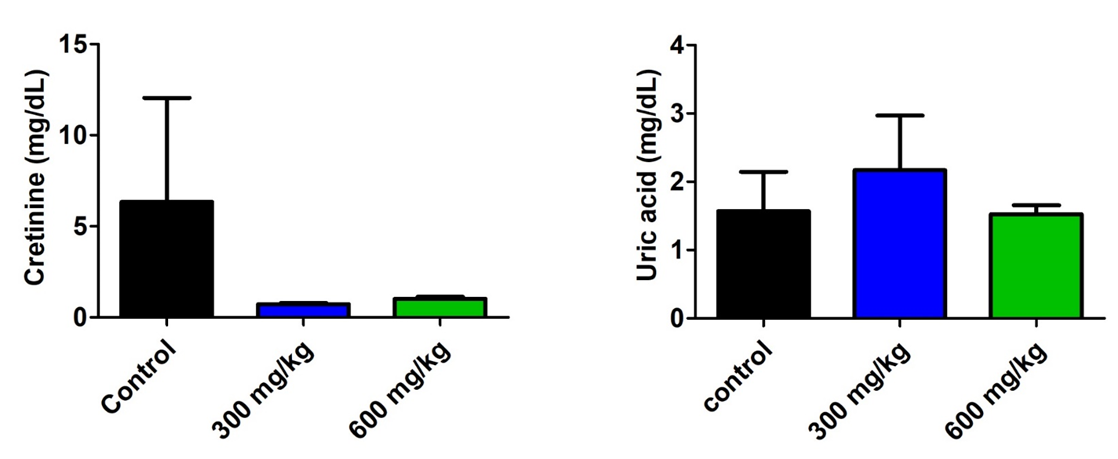


**SUPPORTING INFORMATION FIGURE S2** Effect of Tempeh on the value of serum creatinine and uric acid (mean ± SEM).

**SUPPORTING INFORMATION TABLE S1** Content of total polyphenolic, phenolic compounds and isoflavones in soybean and Tempeh

|  | Soybean | Tempeh |
| --- | --- | --- |
| Total polyphenolic (mg GAE/kg) | 1203 | 6157 |
| Phenolic compounds |  |  |
| Gallic acid (mg/kg) | 1.58 ± 0.44 | 21.70 ± 1.85 |
| Catechin (mg/kg) | 96.34 ± 6.14 | 75.11 ± 2.24 |
| Caffeic acid (mg/kg) | 20.93 ± 2.23 | 16.46 ± 0.45 |
| Rutin (mg/kg) | 11.59 ± 0.89 | 3.88 ± 0.21 |
| Naringin (mg/kg) | 128.86 ± 11.63 | 101.42 ± 5.19 |
| Isoflavones |  |  |
| Daidzin (mg/kg) | 1050 | 667 |
| Genistin (mg/kg) | 948 | 788 |
| Daidzein (mg/kg) | 49 | 420 |
| Genistein (mg/kg) | 63 | 310 |

GAE: gallic acid equivalent.

**SUPPORTING INFORMATION TABLE S2** EC_50_ of antioxidant capacity

|  | EC_50_ of Chelating ability of ferrous ions (mg/mL) | EC_50_ of ABTS radical scavenging ability (mg/mL) |
| --- | --- | --- |
| Soybean | 89.48 ± 4.36 | 215.04 ± 10.42 |
| Tempeh | 6.47 ± 0.14 | 35.90 ± 0.40 |
